# Preliminary Study on Antifungal Mechanism of Aqueous Extract of *Cnidium monnieri* Against *Trichophyton rubrum*

**DOI:** 10.3389/fmicb.2021.707174

**Published:** 2021-08-19

**Authors:** Cao Yanyun, Tang Ying, Kong Wei, Fang Hua, Zhu Haijun, Zheng Ping, Xu Shunming, Wan Jian

**Affiliations:** ^1^Department of Dermatology, Pudong New Area People’s Hospital, Shanghai, China; ^2^Department of Clinical Laboratory, Pudong New Area People’s Hospital, Shanghai, China; ^3^Department of Emergency and Critical Care Medicine, Pudong New Area People’ s Hospital, Shanghai, China

**Keywords:** *Cnidii monnieri*, aqueous extract, *Trichophyton rubrum*, dermatomycosis, chitin synthase

## Abstract

Trichoderma rubrum (T. rubrum) is one of the important pathogens because it is the cause of most dermatomycosis. The treatment of *Trichophyton rubrum* infection is time-consuming and very expensive; it is easy for the infections to reoccur, leading to therapeutic failures, persistence, and chronic infection. These issues have inspired researchers to study natural alternative therapies instead. *Cnidium monnieri* (L.), as a kind of traditional Chinese medicine, has a variety of pharmacological activities and a wide range of applications, so it has a high potential for researching and economic value. We detected the effect of aqueous extract of *C. monnieri* (L.) on the activity of *T. rubrum* by Cell Count Kit-8 assay (CCK-8), and we found that 128 and 256 μg/ml of aqueous extracts of *C. monnieri* (L.) co-cultured with *T. rubrum* for 24 h showed the inhibitory effect on *T. rubrum*. The results of scanning electron microscopy (SEM) and transmission electron microscopy (TEM) confirmed that aqueous extract of *C. monnieri* (L.) damaged the *T. rubrum*. At the same time, mass spectrometry screening with *T. rubrum* before and after the treatment of 256 μg/ml of aqueous extracts of *C. monnieri* (L.) showed that 966 differentially expressed proteins were detected, including 524 upregulated differentially expressed genes (DEGs) and 442 downregulated DEGs. The most significantly downregulated protein was chitin synthase (CHS); and the results of qRT-PCR and Western blotting demonstrated that the expression level of CHS was downregulated in the 256 μg/ml group compared with the control group. The study showed that the aqueous extract of *C. monnieri* (L.) could destroy the morphology of mycelia and the internal structure of *T. rubrum*, and it could inhibit the growth of *T. rubrum*. The antifungal effect of aqueous extract of *C. monnieri* (L.) may be related to the downregulation of the expression of CHS in *T. rubrum*, and CHS may be one of the potential targets of its antifungal mechanism. We concluded that aqueous extract from *C. monnieri* (L.) may be a potential candidate for antifungal agents.

## Introduction

Dermatomycosis is a highly prevalent superficial fungal infection that affects the skin, nails, and hair and is estimated to affect 20–25% of the adult population globally (Seebacher et al., 2008; Zhan and Liu, 2017). It has been observed in different parts of the world. It is an acute and persistent public health problem. *Trichophyton rubrum (T. rubrum)* is the main pathogen of superficial fungal skin infections, accounting for approximately 80–90% of cases (Nenoff et al., 2014). Superficial mycosis generally has mild clinical symptoms and generally affects the quality of life. However, the elderly and those with chronic diseases such as diabetes mellitus (Elewski and Tosti, 2015) and hypoimmune patients have a higher rate of incidence for this disease, and the clinical symptoms may be more serious and even life-threatening (Rouzaud et al., 2015). The treatment of *T. rubrum* infections is time-consuming and very expensive; it is easy for the infections to reoccur. *T. rubrum* may develop drug resistance after a long-term exposure to azole drugs below the inhibitory concentration (Hadrich and Ayadi, 2018), leading to therapeutic failures, persistence, and chronic infection. In addition, several complications, such as bacterial superinfection, may occur (Monod et al., 2019). The causes of unsuccessful treatment also include the susceptibility of the patients, the growth patterns of the drug-resistant fungus, the presence of dormant fungal spores in the affected area, the low bioavailability of drugs, and poor drug penetration (Evans, 2001; Vora et al., 2014; Khamidah and Ervianti, 2018).

These problems have inspired researchers to study natural alternative therapies. Compared with synthetic antibacterial substances, natural plants contain rich biochemical active substances, have broad-spectrum antimicrobial activity, and have a lower toxicity to mammals and less impact on the environment. Drug resistance will not increase with the long-term use of these drugs, which is easy to be widely accepted by the public (Di Vito et al., 2021). On the other hand, the chemical composition of plant extracts determines their medicinal value, and the antibacterial properties largely depend on various factors such as the climatic and geographical conditions, as well as the harvesting, isolation technology, and storage (Helal et al., 2019).

Herbal medicine resources are widely distributed, which plays an important role in a country’s healthcare system. The treatment of fungi with herbal medicine has become increasingly popular throughout the world. Schott (*Dryopteris fragrans*) is traditionally used in northern China to treat fungal infections and other skin diseases (Liu et al., 2018). The inhibitory effect predominantly on *T. rubrum* may be related to the synergistic or cumulative effect of the flavanols distributed on the leaf surface (Gomes et al., 2019). Japanese scholars have found that extracts from different solutions of 15 herbal medicines have an inhibitory effect on *T. rubrum* (Xia et al., 2019). Wu et al. (2008) found that plagiochin E (PLE), an antifungal macrocyclic bis(bibenzyl) isolated from *Rehmannia glutinosa*, affected the synthesis of chitin in the cell wall of *Candida albicans*. The extract of *Scutellaria baicalensis* Georgi, Ou-gong, has an obvious antifungal activity (Da et al., 2019). *Scutellaria barbata* has antibacterial and immunomodulatory effects and a low cytotoxic activity in healthy human bodies (Cordeiro et al., 2021). *Cnidium monnieri* (L.) has an inhibitory effect on *T. rubrum*, *Cryptococcus neoformans*, *Fusarium*, *C. albicans*, *Aspergillus*, and other clinically isolated pathogenic fungi (Jun et al., 2007; Lina et al., 2017).

*Cnidium monnieri* (L.) has a variety of pharmacological activities and a wide range of applications, so it has a high potential for researching and economic value. Coumarins, the main active components of Cnidii Rhizoma, experience a variety of biological activities, including anti-tumor (Shokoohinia et al., 2018), anti-inflammatory (Lee et al., 2014), antispasm (Sadraei et al., 2012), anti-virus (Tamura et al., 2010), and antifungal properties (Matsuda et al., 2002). In order to select effective traditional Chinese medicine preparations based on clinical trial information, Liu et al. (2012) used Chinese e-journal databases to search the relevant Chinese clinical study literature and concluded that *C. monnieri* (L.) should be preferably selected as one of the antifungal plant candidates since they were mostly consistently present in prescriptions or preparations used to treat mycotic vaginitis.

In China, *C. monnieri* (L.) is a compound commonly used with other traditional Chinese medicines such as *Sophora flavescens*, *Phellodendron amurense*, and camphor. Water decoction and tincture are used to treat intractable skin pruritus, eczema, and superficial fungal diseases through fumigation and wet compress. There are few studies on the antifungal mechanism of *C. monnieri* (L.). Previous studies (Wang et al., 2008; Zhao, 2016) showed that the minimum inhibitory concentration (MIC) value of aqueous extract of *C. monnieri* against *Trichophyton rubrum*, *C. albicans*, and *Malassezia* was approximately 5 mg/ml. It is reported that the extract of *C. monnieri* (L.) can enhance the inhibitory effect of macrophages on the *Candida* growth *in vitro* (Matsuda et al., 2002).

In this study, the aqueous extract was obtained directly from the rhizome of *C. monnieri* (L.), *Trichophyton rubrum*, the most common pathogenic fungus, selected for the research. The purpose of this study was to evaluate the inhibitory effect of the aqueous extract of *C. monnieri* (L.) on *T. rubrum*. Scanning electron microscopy (SEM) and transmission electron microscopy (TEM) technologies were used to observe the morphological changes of mycelium. We tried to further explore its antifungal mechanism from proteomics to provide effective targets for the drug research and development involved in treating dermatomycosis.

## Materials and Methods

### Plant Material and Extraction

The aqueous extract of *C. monnieri* (L.) was prepared by decocting 500 g of the dried *C. monnieri* (L.) produced in Shandong Province (purchased from Shanghai Yanghetang Herbal Pieces Co., Ltd., Shanghai, China) with 5 L of boiling distilled water for 2 h, and then it was filtered using filter paper. The aqueous extract was collected in a rotary evaporator and lyophilized, which yielded 75 g of dried powder (yield ratio 15%), and it was stored at −20°C for further use.

### Preparation of Experimental Strains

Twenty strains were isolated from patients with dermatomycosis such as tinea manuum, tinea pedis, and onychomycosis. Then, fungal colonies were identified using a microscope and matrix-assisted laser desorption–time of flight mass spectrometry (MALDI-TOF MS). The isolated *T. rubrum* fungi were inoculated on potato dextrose agar (PDA) plates and cultured at 30°C for 7 days. The fungal suspensions were prepared according to the method of reference (Cao et al., 2020). The fungal suspensions with 0.5 Michaelis concentration were measured by an ultrasonic dispersion counter. The quality control strain was the *T. rubrum* ATCC28188, which was provided by Shanghai Changzheng Hospital.

### Cytotoxicity Assays

The cell viability was determined using the Cell Count Kit-8 assay (CCK-8) (Beyotime Institute of Biotechnology, Shanghai, China). The effect of the aqueous extract of *C. monnieri* (L.) on the activity of *T. rubrum* was detected by the CCK-8. The experimental strains were divided into four groups. The dry powder of *C. monnieri* (L.) was adjusted to 64, 128, and 256 μg/ml concentrations, and *C. monnieri* (L.) aqueous extract was added using RPMI-1640 medium; and these were used as the three experimental groups. The fourth group was the control group and did not add any aqueous extract. In a 96-well cell culture plate, 100 μl of fungal suspension was added at the corresponding position, and 100 μl of the three groups with different *C. monnieri* aqueous extract concentrations was added. The control group was treated with 100 μl of fungal suspension and 100 μl of RPMI-1640 medium. Each sample was repeated three times. After incubation at 28°C for 0.5, 12, and 24 h, the samples were treated in strict accordance with the operation instructions of CCK-8 kit. Ten microliters of CCK-8 solution was added to each well and incubated for another 2 h to measure the cell viability. The optical density of the CCK-8 solution was measured at 450 nm in accordance with the kit instructions.

### Observation of the Ultrastructural Changes of *Trichoderma rubrum* Affected by the Aqueous Extract of *Cnidium monnieri* (L.) Under Electron Microscope

One microliter of *T. rubrum* suspension with a McFarland turbidity of 0.5 was selected and seeded on two Sabouraud dextrose agar (SDA) plates (Shanghai Komajia Microbiology Technology Co., Ltd., Shanghai, China), and each plate was seeded with four spots. The colonies were grown at 28°C for 5 days. Two points were the control group (inoculated with 1 μl of fungal suspension, without any aqueous extract); the other two points will be 1 μl of fungal suspension and 256 μg/ml of aqueous extract of 10 μl, which were mixed and inoculated on SDA plates for 5 days. One plate was used for the electron microscope observation, and the other plate was used for the protein extraction. We selected the control group and the 256 μg/ml group as the observation objects. A small portion of the colony was placed in a sterile 1.5 ml centrifuge tube and fixed with 2.5% glutaraldehyde for 2 h at 4°C. And then after being rinsed with a phosphate-buffered solution three times for 20 min each time, the sample was soaked in 1% osmic acid solution for approximately 2 h at 4°C; dehydrated with 30, 50, 60, 70, 80, 90, 95, and 100% ethanol for 15 min in turn; and then thoroughly dehydrated with 100% ethanol twice for 10 min each time. The samples were dried at the critical point, vacuum coated, observed, and photographed under a SEM (Tecnai G220, FEI, Bionand, Malaga, Spain). After dehydration, the samples were embedded in an epoxy resin, and then ultrathin sections of the sample were sliced after solidification. The other samples were stained with uranium acetate and lead citrate; and they were observed, photographed, and recorded by TEM (Tecnai G220, FEI, Bionand, United States).

### Extraction of the Fungal Protein

After 5 days of culture, the surface of each colony on the SDA plate (Shanghai Komajia Microbiology Technology Co., Ltd.) was gently scraped and centrifuged at 4°C and 3,000 rpm for 5–10 min to collect the fungi. The protein was extracted according to the requirements of the kit (BestBio, Shanghai, China; BB-3136). The fungal cells were washed twice with cold PBS, and the supernatant was drained as much as possible after each washing. A 100-mg sample of ground mycelium powder was put into 1 ml of lysate (20 mmol/L of Tris–HCl, pH 7.4, 150 mmol/L of NaCl, 1% NP40, 0.5% sodium hydroxide, 0.1% sodium dodecyl sulfate (SDS), 1 × Protease inhibitor mixture), and it was incubated on ice for 1 h. During this period, it was mixed upside down every 5 min. It was then centrifuged at 4°C for 30 min at 24,000 rpm.

### Proteomics Mass Spectrometry Analysis

A total of 10 fungal protein samples were analyzed on a hybrid trapped ion mobility spectrometry (TIMS) quadrupole TOF MS (TIMS-TOF Pro, Bruker-Daltonics, Billerica, MA, United States) by CaptiveSpray nano spray ion source. The MS was used in the data correlation mode to generate a spectrum library with enhanced ion mobility. We set the cumulative time and the slope time to 100 ms and recorded the mass spectrum in the range of 100 to 1,700 m/z in the positive electrospray mode. The scanning range of the ion mobility was 0.7–1.3 Vs/cm^2^. The whole acquisition cycle was 1.17 s, including a complete TIMS-MS scan and 10 parallel accumulation–serial fragmentation (PASEF) MS/MS scans. In the PASEF MS/MS scanning process, the collision energy increased linearly with the increase of mobility from 59 eV at 1/K0 = 1.6 Vs/cm ^2^ to 20 eV at 1/K0 = 0.6 Vs/cm^2^.

### RNA Isolation and cDNA Synthesis

Total RNA of *T. rubrum* was extracted from five samples of the 256 μg/ml group and five samples of the control group using the Rnaiso plus kit (Takara Bio, Shiga, Japan) according to the manufacturer’s instructions. The concentration and purity of total RNA were assessed by ultramicro-spectrophotometers (NanoDrop 2000, Thermo Fisher Scientific, Wilmington, DE, United States) at the absorbance ratios of A260/230 and A260/280. Subsequently, the first strand of cDNA was synthesized from 2 μg of total RNA using the FastQuant RT Kit (with gDNase) (Takara Bio, Shiga, Japan) and was stored at −20°C.

### Quantitative Real-Time PCR Detection

Finally, quantification of cDNA levels was carried out following the instructions of SYBR green fluorescence quantitative detection kit (Takara Bio, Shiga, Japan). The primer sequence and product size are shown in Table 1. The reaction process in this study was as follows: pre-denaturation at 94°C for 3 min, 36 repeated cycles for denaturation at 94°C for 30 s and 57°C for 30 s and elongation at 72°C for 1 min, and then maintained at 72°C for 10 min. The whole process of PCR amplification was automatically completed by machine (ABI 7500 Real Time PCR System, ABI, Foster City, CA, United States). The cycle threshold (Ct) value of the target gene (CHS) and internal gene (GAPDH) were obtained, and the relative expression differences for each of the sample were analyzed using the 2^–ΔΔ*CT*^ method (Schmittgen and Livak, 2008).

**TABLE 1:** Primer sequences of qRT-PCR amplification.

**Accession number**	**Target gene primer sequences (5′-3′)**	**Amplification length (bp)**
AB076024	CHS-F GATGACAGTCCGTCCACA	133
	CHS-R GATACAGACTTCAGGGTT	
XM003235298	GAPDH-F ACGGCTTCGGTCGTATTGG GAPDH- R	112
	ATGTATTCGGCGATTTGGTCT	

### Detection of Chitin Synthase Antibody by Western Blotting

The protein of *T. rubrum* was extracted from the control group and the 256 μg/L experimental group by using the above method. The protein content was determined by a bicinchoninic acid (BCA) assay. The proteins were separated by SDS–polyacrylamide gel electrophoresis (PAGE) and then electro-transferred onto polyvinylidene fluoride membranes (Millipore, Billerica, MA, United States). The blocking solution (WB0161) was closed. Anti-CHS (orb242445, rabbit, 1:1,000 solution; Biorbyt, Cambridge, United Kingdom) was incubated overnight at 4°C, and the TBST solution was washed three times. Horseradish peroxidase (HRP)-conjugated antibody goat anti-rabbit IgG H&L (ab6721, 1:2,000; Abcam, Cambridge, MA, United States) was then incubated for 2 h, and the membrane was washed three times after exposure. The relative intensities of the protein bands were measured by the ImageJ image analysis software.

### Statistical Analysis

All data were expressed as the mean ± standard deviation and statistically analyzed by SPSS 21.0 (SPSS Inc., IBM Corp., Armonk, NY, United States). The different groups of data were analyzed using the one-way analysis of variance (ANOVA), and the statistical test level was 0.05. According to the results of the homogeneity test of variance, the least significant difference (LSD) *t*-test was used to test the homogeneity of variance, while Tamhane’s T2 test was used to test the heterogeneity of the variance. A *p* < 0.05 was considered statistically significant.

## Results

### Antibacterial Activity of the Aqueous Extract of *Cnidium monnieri* (L.) With Different Concentrations Against *Trichoderma rubrum*

**FIGURE 1:**
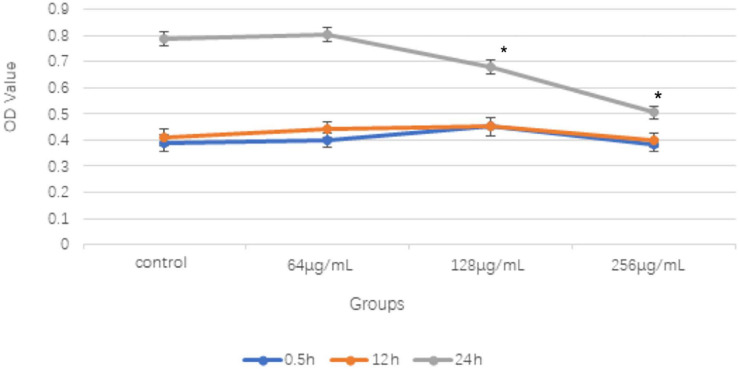
Comparison of OD values of different concentrations of aqueous extract from *Cnidium monnieri* (L.) on *Trichoderma rubrum* in different time periods (x ± s) (*P < 0.05). 128 and 256 μg/mL concentrations of aqueous extract from *C. monnieri* (L.) were used to inhibit *T. rubrum* for 24 h, the OD value difference was statistically significant compared with the control group (*P < 0.05).

Three different concentrations at 64, 128, and 256 μg/ml of the aqueous extract of *C. monnieri* (L.) were co-cultured with *T. rubrum* for 0.5 and 12 h, and there was no significant difference compared with the results of the control group. However, when the three concentrations of the *C. monnieri* (L.) aqueous extract were used to inhibit *T. rubrum* for 24 h, the OD value decreased as the concentrations increased, and the difference was statistically significant compared with that of the control group (Figure 1).

### Electron Microscopic Observations of the Structure of *Trichoderma rubrum* Incubated With Aqueous Extract From *Cnidium monnieri*

**FIGURE 2:**
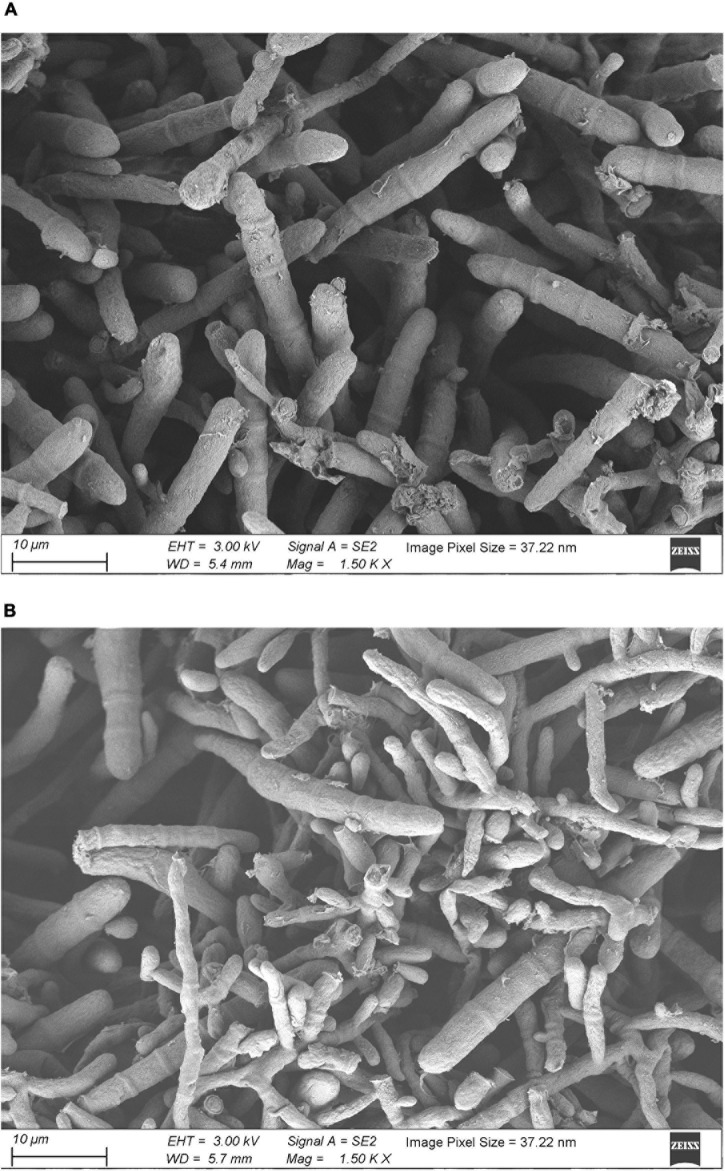
Scanning electron microscopic images of *T. rubrum* of control group and 256 μg/mL group **(A)** control group; **(B)** 256 μg/mL group).

**FIGURE 3:**
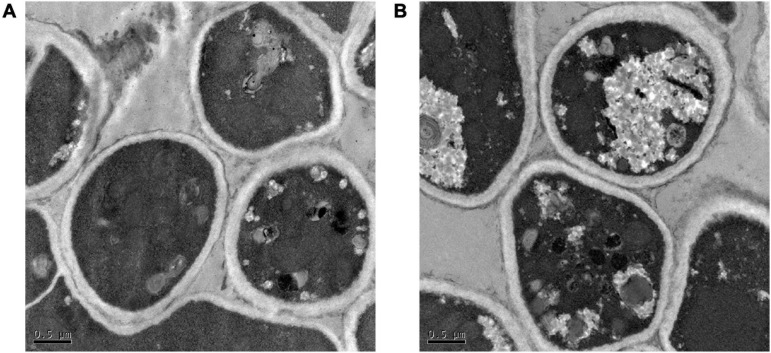
Transmission electron microscopic images of *T. rubrum* of control group and 256 μg/mL group **(A)** control group; **(B)** 256 μg/mL group.

The results of SEM revealed that the control group mycelium had normal shape, a smooth surface, and a uniform thickness (Figure 2A); however, in the 256 μg/ml group, mycelium surface was rough, atrophied, and wrinkled and had different thickness and many damages, fractures, and holes (Figure 2B). Under the TEM, the cell wall of the fungi in the control group was relatively complete, the internal structure was clear, and mitochondria could be seen (Figure 3A). In the 256 μg/ml group, the cell wall was relatively complete, the internal structure had changed significantly, and a large number of structures were destroyed and dissolved (Figure 3B).

### Proteomic Results

**FIGURE 4:**
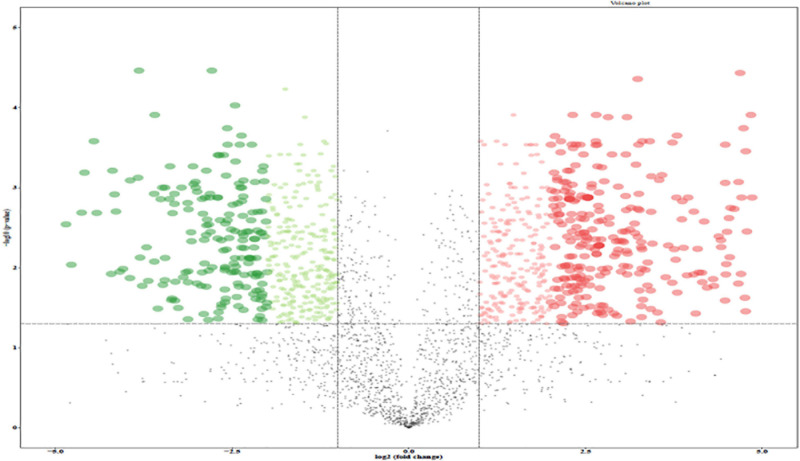
Volcano plot of the differentially expressed genes (DEGs) between control group and 256 μg/mL group. Red dots, green dots, and black dots represent genes with significantly up-regulated expression, significantly down-regulated expression and non-significant difference, respectively.

**FIGURE 5:**
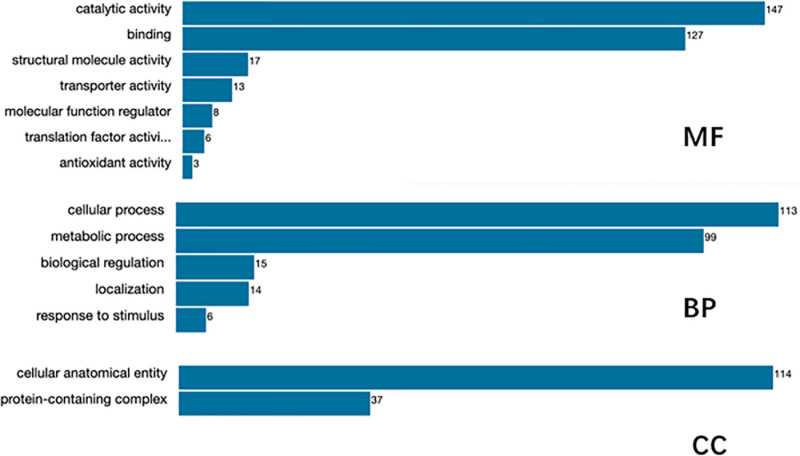
Gene Ontology (GO) function annotation of the identified proteins.

**FIGURE 6:**
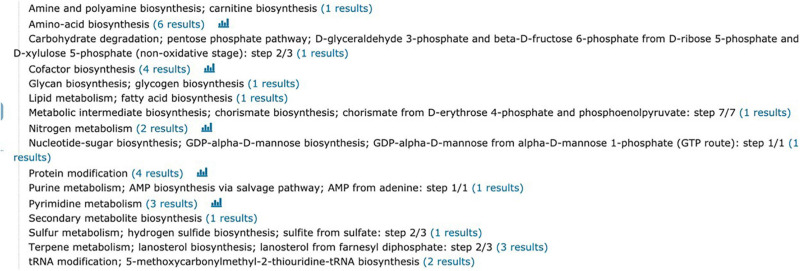
Kyoto Encyclopedia of Genes and Genomes (KEGG) pathway analysis.

A total of 2,634 proteins of *T. rubrum* were identified in all samples. The differentially expressed proteins were screened on the criteria, as follows: fold change > 1.2-fold (upregulated > 1.2-fold or downregulated < 0.83-fold) and *p*-value < 0.05. A total of 966 differentially expressed proteins were detected, including 524 upregulated differentially expressed genes (DEGs) and 442 downregulated DEGs (Figure 4). The top five upregulated proteins are listed in Table 2, and the downregulated proteins are listed in Table 3. Then Gene Ontology (GO) and Kyoto Encyclopedia of Genes and Genomes (KEGG) enrichment analyses were performed on the DEGs with significant difference (Figures 5, 6).

**TABLE 2:** The list of top 5 upregulation proteins between 256 μg/mL group and control group.

**Accession**	**Gene symbol**
A0A178F5P5_TRIRU	NADH-ubiquinone oxidoreductase
A0A178F853_TRIRU	40S ribosomal protein S6
A0A178F4E8_TRIRU	Cystathionine beta-synthase
A0A178ERV4_TRIRU	Coronin
A0A178EU95_TRIRU	Proteasome subunit alpha type

**TABLE 3:** The list of top 5 down-regulation proteins between 256 μg/mL group and control group.

**Accession**	**Gene symbol**
O42708_TRIRU	Chitin synthase
A0A178ENR1_TRIRU	DLH domain-containing protein
A0A178EQZ5_TRIRU	Autophagy-related protein17
A0A178F1S3_TRIRU	Uncharacterized protein
A0A178EVQ6_TRIRU	Uncharacterized protein

Gene Ontology function annotation of the identified proteins was carried out by BLAST2GO software (Figure 5). The annotated candidate proteins are divided into three categories according to their functional characteristics: molecular function (MF), biological process (BP) and cellular component (CC). In the MF, catalytic activity (147 unigenes), binding (127 unigenes), structural molecule activity (17 unigenes), transporter activity (13 unigenes), MF regulator (8 unigenes), and translation factor activity (6 unigenes) were the most abundant groups. Those DEGs were mainly involved in function of the BP, such as cellular process (113 unigenes), metabolic process (99 unigenes), biological regulation (15 unigenes), localization (14 unigenes), and stress response (6 unigenes). The most abundant groups were the cell anatomic bodies and protein-containing complexes in the CC. Another GO category, significant enrichment analysis, was set by Fisher’s exact test to visualize the GO functional annotations and enriched KEGG pathways (Figure 6), Some important BPs showed obvious changes, such as the amino acid biosynthesis, cofactor biosynthesis, protein modification, and pyrimidine metabolism.

### mRNA and Protein Expression Levels of Chitin Synthase

**FIGURE 7:**
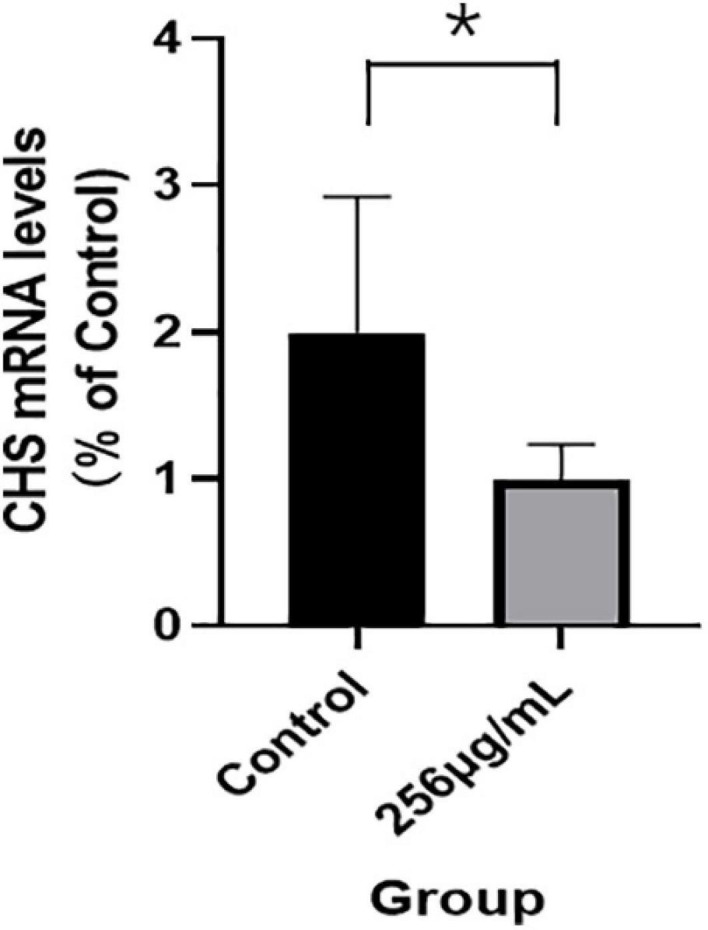
The mRNA expression levels of chitin synthase detected by qRT-PCR (*P < 0.05).

**FIGURE 8:**
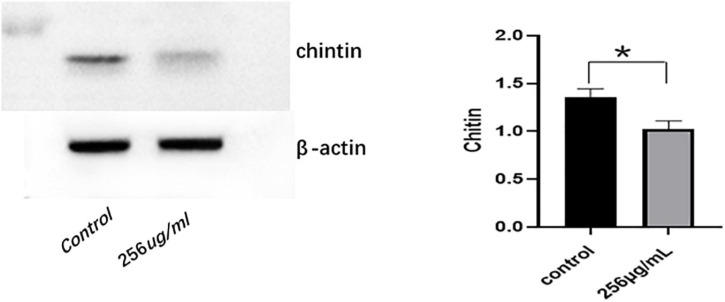
The expression level of chitin synthase (CHS) protein detected by Western blot (*P < 0.05).

The qRT-PCR results indicated that mRNA expression levels of chitin synthase (CHS) in the 256 μg/ml group were lower than those of the control group (*p* < 0.05; Figure 7). As shown in Figure 8, compared with that of the control group, the expression level of CHS was significantly downregulated in the 256 μg/ml group as detected by Western blotting (*p* < 0.05).

## Discussion

*Trichoderma rubrum* is an important pathogen because it causes of most dermatomycosis and is becoming a public health problem, especially when affecting individuals with compromised immune systems (Tullio et al., 2016; Gamage et al., 2020). As an unconditioned pathogen, it is difficult to cure the infection of *T. rubrum*, and it often recurs after drug withdrawal (Kim et al., 2016). Completely removing *T. rubrum* and avoiding its recurrence have always been a difficult problem in treating its infection. At present, topical or oral antifungal drugs are mainly used in the treatment of *T. rubrum*. Existing drugs have their limitations, which include the limited drug types, side effects, and drug resistance. Therefore, there is an urgent need to develop new antifungal drugs with high efficiency, broad spectrum, and selective virulence. Traditional Chinese herbal medicine is rich in compound structure resources, which has become a very important research direction in the search for antifungal drugs with good safety, wide antifungal spectrum, and high efficacy.

Previous studies have shown that the alcohol extract of *C. monnieri* (L.) has a significant inhibitory effect on *T. rubrum*, and the antibacterial rate was enhanced as the drug concentration increased (Lina et al., 2017). In this study, the outcome of the CCK-8 showed that 128 and 256 μg/ml of aqueous extracts of *C. monnieri* (L.) were co-cultured with *T. rubrum* for 24 h, the inhibitory effect on *T. rubrum* was consistent with that of the alcohol extract of *Cnidium ninidium*, and the inhibitory rate was enhanced with the increase of concentration. The results showed that both the water and ethanol extracts of *C. ninidium* had inhibitory effects on *T. rubrum*. However, *Vernonia amygdalina*, *Caulerpa sertularioides*, and *Kappaphycus alvarezii* ethanol extract had an inhibitory effect on *T. rubrum*, and then the aqueous extraction had no obvious inhibition activities (Sit et al., 2018).

To further explore its antifungal mechanism, we selected the aqueous extract of *C. ninidium* with 256 μg/ml concentration and co-incubate it with *T. rubrum* for 5 days. It was observed that the surface of mycelia was rough, atrophied, and uneven in thickness and had multiple damages and fractures with SEM. TEM showed that the cell wall was slightly damaged, and a large number of the internal structures were destroyed and dissolved. This phenomenon indicates that the cell morphology could no longer be maintained and the cell became osmotic and fragile once the continuity of the cell wall was destroyed (Moore et al., 1992). A small damage in the cell wall can result in localized swelling, membrane rupture, and growth inhibition. The results of the SEM and TEM examinations confirmed that the aqueous extract of *C. monnieri* (L.) had a damaging effect on *T. rubrum*. Surprisingly, the results of MS screening of *T. rubrum* before and after the treatment of the drug solution showed that the most significantly downregulated protein was CHS. The mRNA and protein expression levels of CHS were inhibited in the 256 μg/ml group compared with the control group assessed by qRT-PCR and Western blotting.

Chitin synthase is a key enzyme in the chitin synthesis of fungal cell walls, and it is not only involved in the fungi growth and development, host infection, spore formation, and other processes but also closely related to the pathogenicity (Takeshita, 2020). Chitin is indispensable for the construction of the cell wall, and these molecules are usually deeply embedded into the cell wall structure, fixing other components to the cell surface (Brown et al., 2020). The process of *T. rubrum* infection involves the chitin enabling the growth of the fungus and grows hyphae into the toenail. The inhibition of CHS will reduce the proportion of chitin in the cell wall, and the damage to the cell wall and a decrease of electron density can be observed under TEM (Wu et al., 2008). However, our TEM results showed that the cell wall was slightly damaged and that the internal structure of the thallus was obviously damaged. It may be related to many factors such as culture conditions of the fungi, their growth activity, and biological activity of the drugs.

Because chitin is indispensable for the construction of the cell wall of fungi and mammalian cells do not have a cell wall, inhibition of chitin biosynthesis will not cause serious adverse reactions to the human body, which is also a strategy for the research and development of antifungal drugs (Lenardon et al., 2010). The results showed that aqueous extract of *C. monnieri* (L.) inhibited the activity of *T. rubrum*, and the expression of CHS was significantly downregulated. CHS might be one of the potential targets of its antifungal mechanism. The CHS gene affects the fungal growth, cell wall integrity, and toxicity to different extents (Qiu et al., 2021). Therefore, the aqueous extract of *C. monnieri* (L.) may be a potential candidate for safer antifungal agents.

In addition, when combined with common antifungals, natural compounds can minimize the side effects of the dose-related toxicity of these drugs (Zhang et al., 2014) and contribute to the treatment of drug-resistant strains (Ghelardi et al., 2014; Ghannoum, 2016; Danielli et al., 2018; Singh et al., 2018). Coumarin in *C. monnieri* (L.) has recently attracted much attention because it can be used as a common fragment to design new compounds with pharmacological activities (Mladenović et al., 2009; Kurdelas et al., 2010; Ge et al., 2016). For example, researchers have designed novel antifungal CHS inhibitors based on coumarins from *C. monnieri* (L.) that share a side chain with neomycin and polymyxin (Ge et al., 2016). We can also try to modify and transform the chemical structure of the *Cnidium* Cnidii monomer to improve the antimicrobial efficacy of traditional Chinese medicine monomer. In future research, we will continue to expand the sample size of the experiment, to investigate molecular biology technology to conduct more detailed studies on the factors involved in *C. monnieri* (L.) inhibition of *T. rubrum*, and to master the distribution and expression rules of its antimicrobial genes to provide effective targets for the research and development of related drugs.

## Conclusion

The results showed that the aqueous extract of *C. monnieri* (L.) could destroy the morphology of mycelia and internal structure of *T. rubrum* and could inhibit the growth of *T. rubrum*. The antifungal effect of the aqueous extract of *C. monnieri* (L.) may be related to the downregulation of the expression of CHS in *T. rubrum*, and CHS may be one of the potential targets of its antifungal mechanism. We concluded that the water extract from *C. monnieri* (L.) may be a potential candidate for antifungal agents.

## Data Availability Statement

The raw data supporting the conclusions of this article will be made available by the authors, without undue reservation.

## Author Contributions

CY and WJ conceived and designed this study. CY, TY, KW, and ZH collected the data and wrote the manuscript. FH extracted the protein of fungal samples and detected the protein expression level by Western blot. XS, ZP, and WJ analyzed the data and interpreted the results. All authors read and approved the final manuscript and approved the submitted version.

## Conflict of Interest

The authors declare that the research was conducted in the absence of any commercial or financial relationships that could be construed as a potential conflict of interest.

## Publisher’s Note

All claims expressed in this article are solely those of the authors and do not necessarily represent those of their affiliated organizations, or those of the publisher, the editors and the reviewers. Any product that may be evaluated in this article, or claim that may be made by its manufacturer, is not guaranteed or endorsed by the publisher.
